# Improved methodology for assaying brassinosteroids in plant tissues using magnetic hydrophilic material for both extraction and derivatization

**DOI:** 10.1186/1746-4811-10-39

**Published:** 2014-11-24

**Authors:** Jun Ding, Jian-Hong Wu, Jiu-Feng Liu, Bi-Feng Yuan, Yu-Qi Feng

**Affiliations:** Key Laboratory of Analytical Chemistry for Biology and Medicine (Ministry of Education), Department of Chemistry, Wuhan University, Wuhan, 430072 China; Chinese Acad Sci, Key Lab Plant Germplasm Enhancement & Specialty A, Wuhan Bot Garden, Wuhan, 430074 China; College of Chemical Engineering, Wuhan Textile University, Wuhan, 430200 China

**Keywords:** Brassinosteroid, Magnetic solid phase extraction, In situ derivatization, Hydrophilic interaction, Liquid chromatography-mass spectrometry

## Abstract

**Background:**

Brassinosteriods (BRs) are a group of important phytohormones that have major effects on plant growth and development. To fully elucidate the function of BRs, a sensitive BR assay is required. However, most of the previously reported methods are tedious and time-consuming due to multiple pretreatment steps. Therefore, it is of great significance to develop a method to increase the throughput and detection sensitivity of BR analysis.

**Results:**

We established a novel analytical method of BRs based on magnetic solid phase extraction (MSPE) combined with in situ derivatization (ISD). TiO_2_-coated magnetic hollow mesoporous silica spere(TiO_2_/MHMSS) was served as a double identity- a microextraction sorbent and “microreactor” for the capture and derivatization of BRs in sequence. BRs were first extracted onto TiO_2_/MHMSS through hydrophilic interaction. The BR-adsorbed TiO_2_/MHMSS was then employed as a “microreactor” for the derivatization of BRs with 4-(N,N-dimethyamino)phenylboronic acid (DMAPBA). The MSPE-ISD method was simple and fast, which could be accomplished within 10 min. Furthermore, the derivatives of BRs showed better MS response because they were incorporated with tertiary amino groups. Uniquely, endogenous BRs were detected in only 100 mg fresh weight plant tissue.

**Conclusion:**

Our proposed MSPE-ISD method for the determination of endogenous BRs is rapid and sensitive. It can be applied to the analysis of endogenous BRs in 100 mg fresh plant tissue (*Brassica napus* L. (*B. napus* L)). The proposed strategy for plant sample preparation may be extended to develop analytical methods for determination of a wide range of analytes with poor MS response in other complex sample matrices.

## Background

Brassinosteroids (BRs), a class of polyhydroxy steroid phytohormones, play critical roles in the growth and development of plants, including the germination of seeds, rhizogenesis, flowering, senescence, photomorphogenesis etc. [1, 2]. Extensive studies also suggest that BRs can synergize with other phytohormones to function in the processes of reproduction, embryogenesis, hypocotal elongation and so on [3–5]. The investigations of BR functions rely heavily on monitoring of the temporal and spatial variation of the BR concentrations. Therefore, an effective BR analytical method is necessary.

In recent years, the technological breakthroughs in instrumentation have improved the selectivity and sensitivity of analytical methods with the advent of high-performance liquid chromatography-tandem mass spectrometry (HPLC-MS/MS) [6]. However, for the analysis of plant samples, the compromised sensitivity is frequently caused by the signal suppression from complex sample matrix during mass spectrometry (MS) analysis. Moreover, the trace amounts of BRs in complex plant matrixes and their inherently low MS response makes reliable qualitative and quantitative analysis of BRs challenging. The current pretreatment methods of BRs to remove the sample matrix required the combination of two or more sample preparation processes, including SPE [7, 8], LLE [9], MSPE [10] etc. Besides, BRs lack ionization groups, thus the MS responses of BRs are far from satisfaction. To improve MS responses of BRs, a pre-column derivatization process was employed to incorporate ionized moieties into BRs before LC-MS analysis [7, 11]. Obviously, the multiple sample preparation processes with the following derivatization procedure made BR analysis labor-consuming and time-consuming. Therefore, it is essential to develop a fast and sensitive BR assay.

In situ derivatization (ISD) is a relatively new technique, which can couple with multiple sample preparation methods to simplify the connection of the extraction and derivatization [12, 13]. So far, single-drop microextraction (SDME) [14, 15], solid-phase extraction (SPE) [16, 17], hollow fiber liquid–liquid–liquid extraction (HF-LLLME) [18, 19], polymer monolith microextraction (PMME) [20], solid phase microextraction (SPME) [21, 22] and stir bar sorptive extraction (SBSE) [23], have effectively combined with ISD for the analysis of a variety of compounds. Herein, the extraction media served as a double identity—an extractant and microreactor. After analytes were loaded onto the extraction media, the chemical derivatization reaction can occur directly on the surface of the sorbents by adding derivatization reagent. In the process, a redundant desorption/re-dissolution step was prevented and the errors associated with the multi-step sample preparation process were reduced. Most importantly, the enrichment of target analytes in the extractant would benefit the fast derivatization reaction due to the local relatively high concentration. Despite of the advantages of ISD, considerable pretreatment time was still required to achieve satisfactory extraction efficiency due to the inherent limitation of the current extraction methods themselves.

Magnetic solid phase extraction (MSPE), a new mode of extraction technique based on magnetic or magnetizable nanoparticles, has been widely used in sample preparation in recent years [24–27]. The sorbents can be dispersed uniformly in sample solution by vortex, instead of being packed into the SPE cartridge. Moreover, magnetic sorbents can be readily agglomerated and re-dispersed in a sample solution by the application and removal of an external magnetic field, which makes the phase separation very convenient. From the view of mass transfer, the dispersive extraction mode also provides a large contact area between the extractant phase and sample solution, which is favorable for the mass transfer of analytes and therefore results in shorter extraction time [28]. In virtue of these properties, MSPE coupled with ISD is a promising technique for the fast and sensitive pretreatment of BRs.

BRs contain multiple polyhydroxy groups and thus exhibit hydrophilic property. In light of this property, hydrophilic magnetic materials were chosen as sorbents, and a fast and convenient MSPE-ISD method based on hydrophilic interaction was developed for the determination of endogenous BRs in plant tissues. By employing hydrophilic magnetic material as both a microextraction sorbent and “microreactor”, the MSPE-ISD method integrates extraction and derivatization together, which largely simplifies the analytical process. First, BRs were extracted onto the surface of a magnetic sorbent through hydrophilic interaction in the acetonitrile extract of the plant sample; in the meantime, hydrophobic interferents from the extract were removed. Subsequently, magnetic sorbents served as a “microreactor”, where the captured BRs were rapidly and efficiently derivatized with 4-dimethylphenyl boronic acid (DMPBA). The BR derivatives could be desorbed from the sorbents with water as the desorption solvent for further UPLC-ESI-MS/MS analysis. The proposed MSPE-ISD procedure could be accomplished within 10 min, and endogenous BRs could be detected in 100 mg fresh weight plant tissues.

## Results and discussion

### Optimization of MSPE-ISD

The proposed MSPE-ISD method for the analysis of BRs utilized hydrophilic interaction to fulfill both the extraction and ISD process. In hydrophilic interaction chromatography (HILIC), the high content of acetonitrile is normally used as the sampling solution. It was already reported that the extraction efficiencies of BRs in acetonitrile were satisfactory [29], which provides an opportunity to separate them from the hydrophobic interferents based on hydrophilic interaction. Moreover, the cis-diol groups in the BR structure can react efficiently with boronate derivatization reagent [10, 30]. Based on these backgrounds, a series of magnetic hydrophilic materials were chosen as sorbents, and DMAPBA was selected as the derivatization reagent. Several parameters affecting the extraction and derivatization efficiencies were investigated.

**Figure 1:**
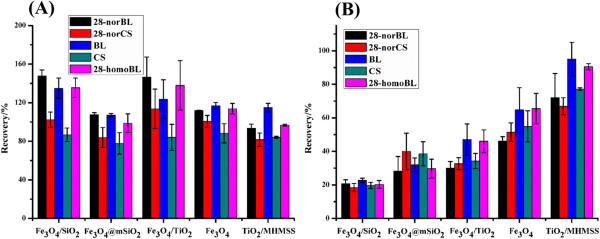
**Effect of different magnetic sorbents on the BR recoveries in acetonitrile solution sample (n = 2) (A) and plant extract (n = 3) (B).** BRs were spiked in the sampling solution at 1 ng/mL each.

We first examined the performance of different types of sorbents on the extraction of BRs. Plant extract with acetonitrile contains large amounts of interfering matrix, such as pigments and hydrophobic compounds, which may jeopardize the following in situ derivatization and UPLC-ESI-MS/MS analysis. As BRs exhibit relative hydrophilic properties due to their multiple hydroxyl groups, we selected magnetic hydrophilic sorbents (TiO_2_/MHMSS, Fe_3_O_4_, Fe_3_O_4_@mSiO_2_, Fe_3_O_4_/SiO_2_, Fe_3_O_4_/TiO_2_) for the extraction of BRs and the removal of hydrophobic matrix and pigments in the hydrophilic solid-phase extraction mode. Comparison of the performance of these hydrophilic sorbents on the extraction of BRs was conducted by examining the recoveries of BRs in acetonitrile or in plant extract. As shown in Figure 1, the five types of hydrophilic sorbents exhibited no significant difference in the recoveries of BRs spiked in acetonitrile (Figure 1A), whereas remarkable differences in the recoveries of BRs spiked in the plant extract were observed (Figure 1B). The extraction efficiencies for BRs are in the order of TiO_2_/MHMSS > Fe_3_O_4_ > Fe_3_O_4_/TiO_2_ > Fe_3_O_4_@mSiO_2_ > Fe_3_O_4_/SiO_2_, which may be ascribed to their differences in hydrophilic properties and the number of adsorption sites for BRs and polar compounds from the plant matrix. For BRs spiked in acetonitrile, all the five sorbent exhibited great extraction efficiencies towards BRs due to no matrix effect. However, for the plant extract, the massive matrix interferents competed with BRs for the adsorption sites, leading to low extraction efficiencies of sorbents to different extent. To assure sufficient recoveries of BRs, TiO_2_/MHMSS was chosen as the hydrophilic sorbent for the following experiments.

**Figure 2:**
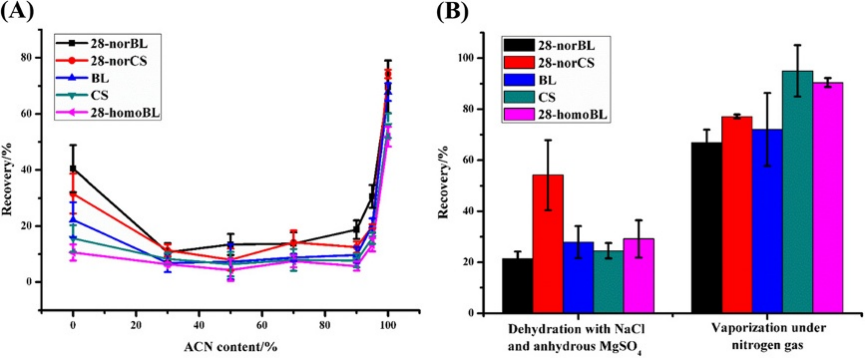
**Effect of the water content in the sampling solution on the BR recoveries (n = 3) (A) and comparison of the effects of different dehydration strategies on plant extract (n = 3) (B).** BRs were spiked in the sampling solution at 1 ng/mL each.

We further optimized the sampling solution in MSPE-ISD. Volume percentages of acetonitrile in the range of 0 to 100% were investigated. As shown in Figure 2A, the highest recoveries were obtained with 100% acetonitrile; once, the proportion of water was greater than 5% (v/v), the recoveries dropped dramatically, suggesting that the extraction efficiencies of BRs by hydrophilic sorbent of TiO_2_/MHMSS are strongly dependent on the acetonitrile content (more than 95%) in the sampling solution.

To obtain high extraction efficiencies of BRs by TiO_2_/MHMSS, the water in the extraction solution should be removed as much as possible. However, in the process of the grinding of plant tissues in liquid nitrogen and acetonitrile extraction, water was inevitably brought into the plant extract. Here, we evaluated the dehydration strategies by either direct evaporation of plant extract and reconstitution with acetonitrile or the addition of NaCl and anhydrous MgSO_4_ into the plant extract. As shown in Figure 2B, a better dehydration effect was obtained by direct evaporation of plant extract and reconstitution with acetonitrile. Hence, the plant extract was evaporated under a mild nitrogen atmosphere and then re-dissolved in acetonitrile for the following experiments.

**Figure 3:**
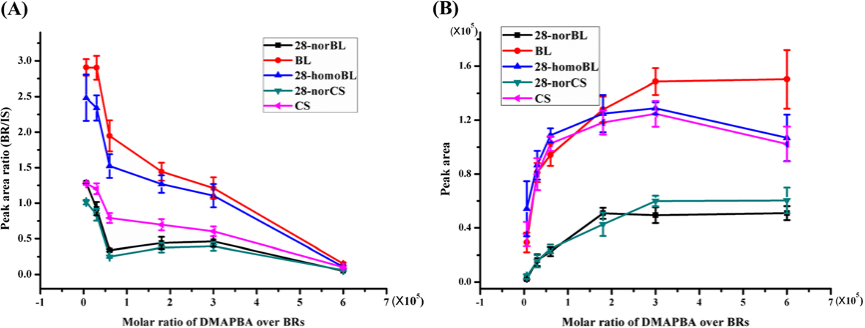
**Effect of the DMAPBA amount on the derivatization efficiencies of BRs in acetonitrile (n = 3) (A) and in plant extract (n = 3) (B).** BRs were spiked in the sampling solution at 1 ng/mL each.

Because the plant extract was very complex, DMAPBA would also react with cis-diol-containing interferents in the plant extract. Therefore, to ensure high derivatization efficiency, the DMAPBA amount was investigated in both acetonitrile solution spiked with BR standards and plant extract spiked with BR standards (1 ng for each) (Figure 3). In acetonitrile, the peak areas of the BR derivatives dropped as the molar ratios increased (Figure 3A), whereas in the plant extract, the maximal peak areas appeared at molar ratios (DMAPBA molar quantity by five BR molar quantity) above 300,000 (Figure 3B). We reason that in acetonitrile solution, as the molar ratio increased, more DMAPBA would enter the LC-MS/MS system during sample injection, which may suppress the ionization efficiencies of the BR derivatives. In plant extract, the existing BR analogues and other cis-diol-containing compounds might consume large amounts of DMAPBA; therefore, a greater amount of DMAPBA was required to guarantee high derivatization efficiencies of BRs. In this regard, 500 μg DMAPBA (molar ratio 500,000/1) was selected.

**Figure 4:**
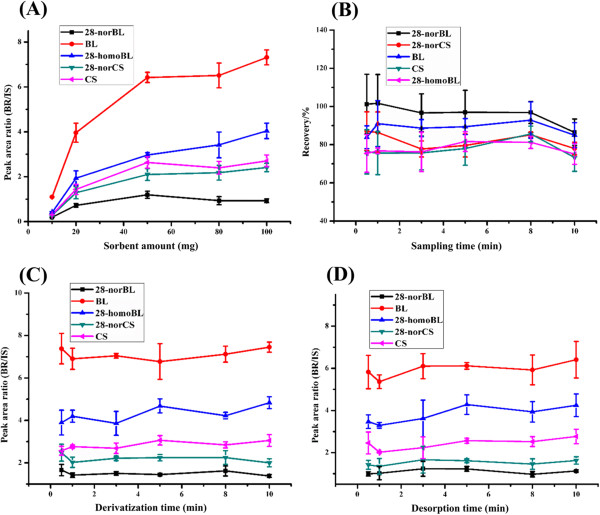
**Effect of the sorbent amount on the extraction and derivatization efficiencies (n = 3) (A), effects of extraction time (n = 3) (B), derivatization time (n = 3) (C) and desorption time (n = 3) (D) on the extraction and derivatization efficiencies.** BRs were spiked in the sampling solution at 1 ng/mL each, and DMAPBA (500 μg/mL) was added for derivatization.

TiO_2_/MHMSS amounts were examined in the range of 10 to 100 mg. As shown in Figure 4A, the signal of the BR derivatives significantly increased as the TiO_2_/MHMSS amounts increased from 10 mg to 50 mg, and most of the signal of the BR derivatives remained nearly constant with greater amounts of TiO_2_/MHMSS. Therefore, 50 mg TiO_2_/MHMSS sorbent was used in the following experiments.

To obtain fast mass transfer between the sorbent and plant extract, the sampling, derivatization and desorption process were all performed under vortexing. Sampling time ranging from 30 seconds to 10 minutes was investigated. As shown in Figure 4B, the sampling time had no obvious effect on the extraction efficiencies; therefore, 30 seconds sampling was chosen. Similarly, the derivatization time and desorption time were also optimized (Figure 4C and D). The results showed that 30 seconds was enough for both procedures.

On the basis of the above-described discussion, the optimal extraction conditions were as follows: 50 mg TiO_2_/MHMSS as the sorbents, BRs in acetonitrile (1 mL) as the sampling solution, 500 μg/mL DMAPBA in acetonitrile (1 mL) as the derivatization solution, H_2_O (0.5 mL) as the desorption solution, 30 s for the extraction, derivatization and desorption time. In the optimal conditions, the MSPE-ISD process could be accomplished within 10 minutes.

### Sensitivity evaluation

**Figure 5:**
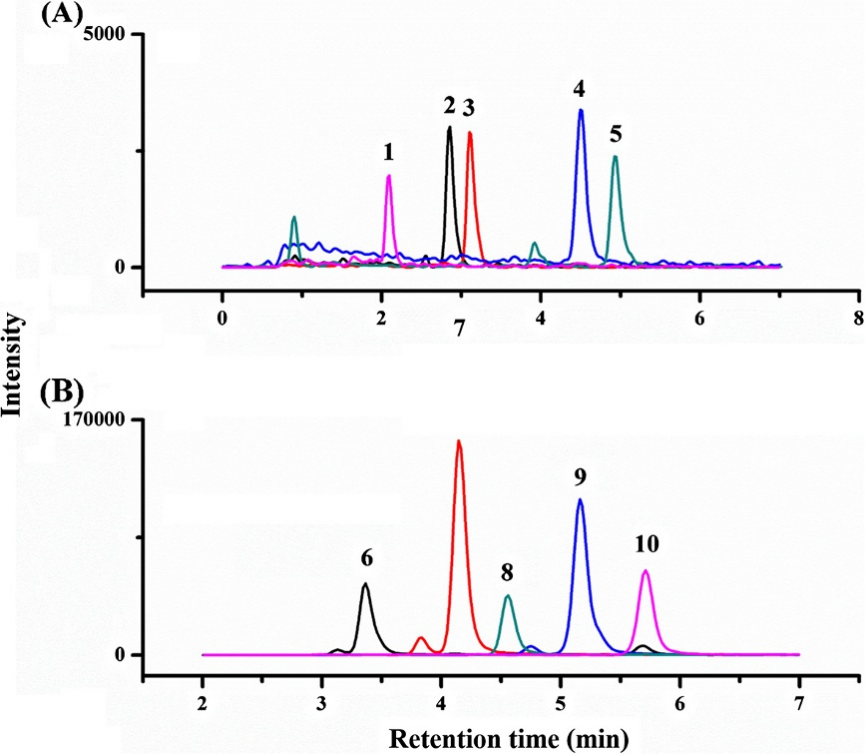
**MRM chromatograms of the five BRs obtained without (A) or with MSPE-ISD (B).** Peaks: 1. 28-norBL; 2. 28-norCS; 3. BL; 4. CS; 5. 28-homoBL; 6. 28-norBL derivative; 7. BL derivative; 8. 28-norCS derivative; 9. 28-homoBL derivative; 10. CS derivative.

To evaluate the performance of MSPE-ISD, we compared the detection sensitivity of BRs with or without MSPE-ISD. After treatment, equal amount of BRs or BR-derivatives were injected into LC-MS/MS system. As shown in Figure 5, significant enhancement of the peak areas of five BRs could be achieved by MSPE-ISD. Specifically, after labeled with DMAPBA by ISD method, the peak areas of the BR derivatives increased by 18-48-fold compared to that of BRs, which demonstrated the MS responses of BRs greatly increased.

### Method validation

**Figure 6:**
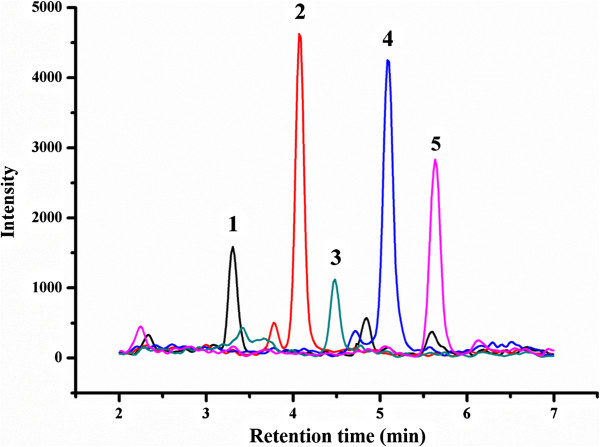
**MRM chromatograms of the BRs in plant tissue (100 mg FW) treated with the MSPE-ISD method.** The five BRs were all spiked at 0.1 ng/mL. Peaks: 1. 28-norBL; 2. BL; 3. 28-norCS; 4. 28-homoBL; 5. CS.

Because only two IS standards are commercially available and this is not sufficient to normalize the extraction and derivatization process, matrix-matched calibration curves were chosen as reference curves in the current study. The calibration curves were constructed by plotting the analyte/IS peak area ratio versus the concentrations with triplicate measurements from 100 mg rice shoots. MRM chromatograms of the BRs in plant tissue spiked at 1 ng/g are shown in Figure 6. As shown in Table 1, satisfactory correlation coefficients were obtained with R values ranging from 0.9867 to 0.9992. Moreover, the sensitivity of the method was evaluated by examining the limit of detection (LOD) and the limit of quantification (LOQ). The LOD was defined as the lowest detectable concentration with a signal-to-noise ratio of at least 3, and the LOQ was defined as the lowest quantifiable concentration with a signal-to-noise ratio of at least 10. The LODs and LOQs were in the range of 1.94 to 5.12 ng/L and 6.48 to 17.07 ng/L, respectively.

**Table 1:** **Linearities, LODs and LOQs of the BR derivatives**

Analyte	Linear range	Regression data			LODs	LOQs
	(ng/mL)	Slope	Intercept	R value	(ng/L)	(ng/L)
28-norBL	0.01-5	6.9291	0.0448	0.9867	4.86	16.20
BL	0.01-5	9.6684	0.0560	0.9981	1.94	6.48
28-homoBL	0.01-5	7.4722	0.0669	0.9923	4.49	14.97
28-norCS	0.01-5	6.0189	0.0262	0.9872	5.12	17.07
CS	0.01-5	7.5004	-0.0103	0.9992	4.23	14.08

The reproducibility and accuracy of the proposed method were evaluated by intra- and inter-day precisions and recoveries. *O. sativa* L shoot extracts were spiked with BR standards (BL, CS, 28-norBL, 28-norCS, and 28-homoBL) at three concentration levels (0.5 ng/g, 1 ng/g, and 10 ng/g). Three parallel extractions of a sample solution over 1 day gave the intra-day RSDs, and the inter-day RSDs were determined by extracting sample solutions that had been independently prepared for 3 continuous days. As shown in Table 2, acceptable precision was obtained, with RSD values below 16.3%, indicating good reproducibility of the proposed method.

**Table 2:** **Accuracy and precision (intra- and inter-day) for the determination of BRs in** ***O. sativa*** **L seedlings (100 mg FW)**

Analyte	Intra-day precision (RSD, %, n = 3)			Inter-day precision (RSD, %, n = 3)			Recovery (%, n = 4)		
	Low	Medium	High	Low	Medium	High	Low	Medium	High
	(0.5 ng/g)	(1 ng/g)	(10 ng/g)	(0.5 ng/g)	(1 ng/g)	(10 ng/g)	(0.5 ng/g)	(1 ng/g)	(10 ng/g)
28-norBL	7.6	12.4	0.4	12.6	14.7	5.7	115.4	109.7	111.3
BL	5.7	7.4	8.0	1.3	10.5	4.1	102.9	111.0	113.7
28-homoBL	14.6	8.3	0.0	16.0	14.0	16.3	113.0	120.9	119.7
28-norCS	10.9	7.7	3.9	8.6	10.0	14.5	110.6	94.2	96.4
CS	12.5	7.5	5.8	2.1	11.7	8.0	109.1	108.0	112.9

The recoveries were also obtained using *O. sativa* L extracts. The endogenous concentrations of BRs in *O. sativa* L extract were calculated based on the calibration curves. The spiked BR amounts were calculated by subtracting the endogenous concentration of each BR in the extract from the total concentration of BRs. Therefore, the recoveries were obtained by comparing the concentration of measured spiked BRs with the corresponding spiked values. As shown in Table 2, the relative recoveries were in the range of 94.2% to 119.7%, demonstrating that the accuracy of the proposed method was satisfactory.

### Effect of plant tissue amount on BR detection

**Figure 7:**
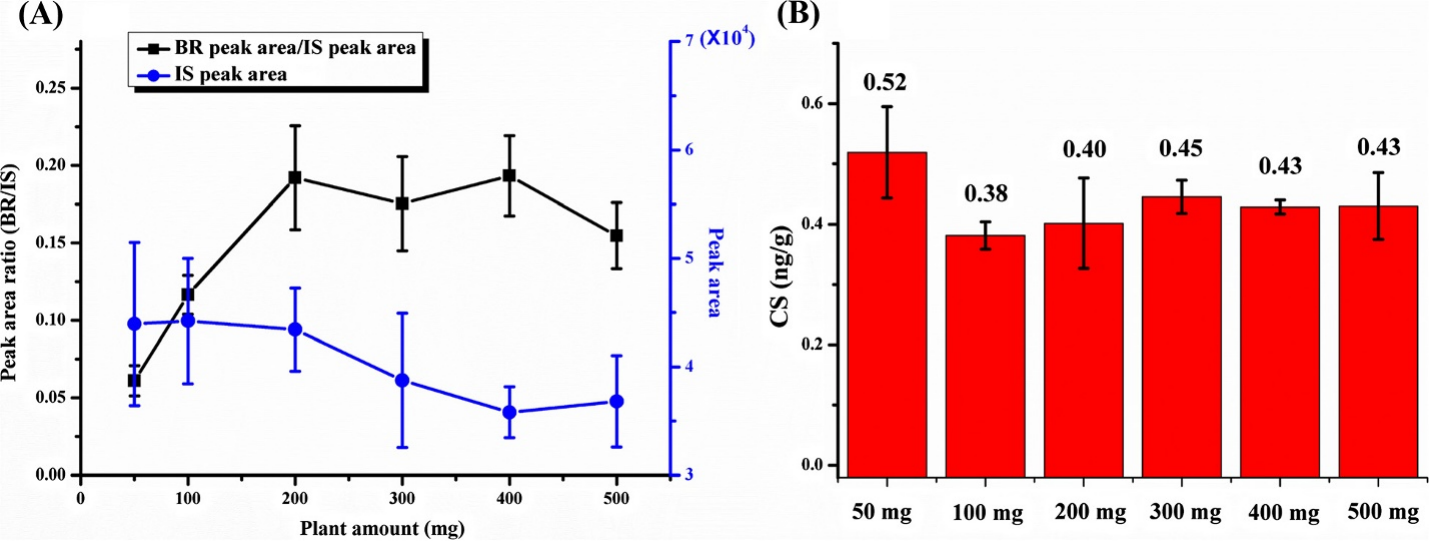
**Effect of plant tissue amount on BR assay.** Effect of the plant amount on the extraction efficiencies (black line) and mass response (blue line) (n = 3) **(A)**. Investigation of the minimal amount of plant tissue (n = 3) **(B)**. O. sativa L shoot was analyzed.

With increased amounts of plant tissue, the endogenous BR contents also increased, which would facilitate BR detection. However, increased amounts of plant tissue may introduce more matrix interferents and therefore cause a negative impact on both extraction and detection. In this vein, an appropriate sample amount should be selected. Different amounts of plant tissue (50-500 mg) were treated by the MSPE-ISD method, and IS derivatives were added prior to the UPLC-ESI-MS/MS analysis (Figure 7A). When matrix effects are negligible, the peak area of the IS derivatives should keep constant with the increase of plant amount, and the ratio of BR peak area to IS derivative peak area should increase linearly with the increase of plant amount. However, the matrix effects on the extraction and detection were obviously observed when using plant samples greater than 100 mg. The matrix effect of 100 mg of plant tissue was 67.4 to 93.1%, indicating that most of the hydrophobic matrix that might have a negative effect on ESI-MS ionization of BR derivatives had been removed using 100 mg plant tissue (Table 3).

**Table 3:** **Matrix effect of plant tissue analyzed by MSPE-ISD**

Analytes	Matrix effect/%
28-norBL	85.9
BL	67.4
28-homoBL	76.0
28-norCS	93.1
CS	77.8

Matrix effect = BR/IS peak area ratio of the real sample over the BR/IS peak area ratio of a standard sample.

Plant extract (100 mg FW) and acetonitrile were both spiked with five BRs at 0.1 ng/mL.

In some cases, a limited amount of plant tissue can be obtained for phytohormone analysis. To investigate the minimal amount of plant tissue required for endogenous BR detection, different amounts (from 50 to 500 mg) of *O. sativa* L shoots were used for the analysis of endogenous BRs by the MSPE-ISD method. As shown in Figure 7B, the results showed that the quantification of endogenous BRs was not affected by different amounts of *O. sativa* L shoot, but the signal-to-noise ratio of CS was near the LOQ when the amount was less than 50 mg. Therefore, 100 mg was used for the real sample analysis.

### Analysis of BRs in plant tissues

The BR contents in five plant samples (the control and drought *O. sativa* L shoot, *O. sativa* L. cv. 9311-A shoot, *O. sativa* L. cv. 9311-B shoot and *Brassica napus* L. shoot) were determined by the MSPE-ISD method. The results showed that both CS and BL were detectable in *Oryza sativa* L. (control) and *Brassica napus* L. shoots, and CS was detected in *Oryza sativa* L. (drought), *Oryza sativa* L. cv. 9311-A and *Oryza sativa* L. cv. 9311-B shoots (Table 4), which demonstrates that our proposed method is suitable for the sensitive analysis of low contents of BRs in plant tissue.

**Table 4:** **Amounts of endogenous BR in various plant tissues**

Analyte	***O. sativa***L. (control)	***O. sativa***L. (drought)	***B. napus***L. shoot	***O. sativa***YTA shoot	***O. sativa***YTB shoot
CS	0.09 ± 0.01	0.11 ± 0.04	0.17 ± 0.02	0.19 ± 0.02	0.26 ± 0.02
BL	0.04 ± 0.01	n.d.	0.13 ± 0.01	n.d.	n.d.

Unit: ng/g; n.d., not detectable.

Furthermore, we designed a biological experiment to test the proposed method. BRs were reported to take part in plant photomorphogenesis [31]. In light, the related genes in BR biosynthesis pathway were inhibited, while in dark these genes got activated. To investigate the effect of light periods on the BR levels, we grew *O. sativa* L under three different light periods (all dark, 8 h light/16 h dark, 16 h light/8 h dark) and observed different growth patterns of these *O. sativa* L shoots. In dark, the seedlings showed an etiolation pattern that did not produce chlorophyll but instead elongated upwards. In light, the seedlings were all green and relatively short. The endogenous BR contents of the three samples were analyzed by our proposed method. As shown in Table 5, the BR contents showed no difference between the seedlings of the 8 h light/16 h dark and 16 h light/8 h dark conditions. Remarkably, the CS content was reduced sharply in the all dark condition, whereas 0.04 ng/g BL was observed. BL was the final product of the BR biosynthesis pathway and was reported to be the most active among all of the BRs. The quantitative results of BRs revealed that BR synthesis gene got activated, and CS was converted into BL in the absence of light, which was coincided with the reported physiological function of BRs, demonstrating the feasibility and accuracy of the proposed BR assay.

**Table 5:** **Amounts of endogenous BR in** ***O. sativa*** **L shoots under three different light conditions**

Analyte	***O. sativa***L shoot with 16 h light/8 h dark	***O. sativa***L shoot with 8 h light/16 h dark	***O. sativa***L shoot with all dark
CS	0.10 ± 0.02	0.12 ± 0.01	0.04 ± 0.00
BL	n.d.	n.d.	0.04 ± 0.01

Unit: ng/g; n.d., not detectable.

### Method comparison

We summarized the representative articles published in the last four years for BR analysis using different methods in Table 6 [7, 9, 30, 32], and the analytical time, LODs and the amount of samples were compared. The proposed MSPE-ISD-UPLC-MS/MS assay could be finished within an hour, and only 100 mg fresh weight of plant tissues were required for the quantification of endogenous BRs. Compared with the published methods, the proposed method showed significant advantages in both the sensitivity and the analysis speed.

**Table 6:** **Comparison of different BR analytical methods**

Pretreatment method	Separation/detection	Analyte	LOD	Amount of plant tissues	Analysis time
LLE-MSPE-derivatization [9]	LC-FLD	24-epiBL	0.12 ng	50 g	More than 3 hours
SPE-ultrafiltration-SPE-derivatization [7]	Online trapping-UPLC-MS/MS	28-epihomoBL	0.2 pg	400 mg	7 hours
MCX SPE-MAX SPE-derivatization [32]	UPLC-MS/MS	BL, CS, teasterone (TE), typhasterol (TY)	1.5-3.9 pg	1 g	1 day
On-line two-dimensional microscale SPE-on column derivatization-HPLC-MS/MS [30]	On-line-HPLC-MS/MS	24-epiBL, 24-epiCS, 6-deoxo-24-epiCS,TE, TY	1.4-6.6 pg	225 mg	40 minutes
MSPE coupled with ISD (this work)	UPLC-MS/MS	28-norBL, 28-norCS, 28-homoBL, BL, CS	0.1-0.3 pg	100 mg	1 hour

## Conclusion

In this study, we developed an MSPE-ISD method for the determination of endogenous phytohormones in plant tissues. Using TiO_2_/MHMSS as both an extraction sorbent and microreactor, the extraction and derivatization processes and magnetic separation were successfully combined. The method largely simplified the sample preparation procedure and the BR assay can be accomplished within 1 hour. In the meantime, the MS response of BRs was significantly improved due to derivatization with 4-DMAPBA, which can benefit the quantification of BRs with a small amount of plant tissue (100 mg fresh weight in the current study). We then successfully determined the concentration of endogenous BRs in various plant tissues. The developed MSPE-ISD technique may also have potential for the determination of a wide range of analytes in other complex biological and environmental sample matrices.

## Methods

### Chemicals and reagents

Standard BRs and stable isotope-labeled standards (IS), including 28-norbrassinolide (28-norBL, purity > 98%), 28-norcastasterone (28-norCS, purity >98%), 28-homobrassinolde (28-homoBL, purity >95%), brassinolide (BL, purity >95%), castasterone (CS, purity >98%), [^2^H_3_]BL and [^2^H_3_]CS, were purchased from Olchemim Ltd. (Olomouc, Czech Republic). All of the BRs standards and stable isotope-labeled standards were dissolved in acetonitrile to obtain stock solutions at the concentration of 200 ng/mL for each. Working solutions were obtained by appropriate dilution of the stock solutions.

Chromatographic grade acetonitrile was obtained from Tedia Co. (Fairfield, OH, USA). Ultrapure water was purified by a Milli-Q water purification system (Millipore, Milford, MA, USA). 4-(N,N-dimethyamino) phenylboronic acid (DMAPBA) was purchased from J&K Scientific Ltd (Beijing, China). Cetyltrimethylammonium bromide (CTAB), sodium silicate nonahydrate (Na_2_SiO_3_ · 9H_2_O), iron nitrate nonahydrate (Fe(NO_3_)_3_ · 9H_2_O), ethylene glycol (EG), ammonium hexfluorotitanate ((NH_4_)_2_TiF_6_), boric acid (H_3_BO_3_) and ethyl acetate were all of analytical grade and supplied by Sinopharm Chemical Reagent Co., Ltd (Shanghai, China). Titania spheres (Titansphere, 5 μm) were purchased from GL Sciences Inc. (Tokyo, Japan). Silica spheres (SiO_2_, 200-300 mesh) were obtained from Qingdao Haiyang Chemical Co., Ltd (Qingdao, China).

### Plant materials

Nine types of plant leaves, including rice (*Oryza sativa* L. (*O. sativa* L)) and rape (*B. napus* L), were analyzed in this study. Three-month-old wild-type B. napus L leaves were harvested from the ground. Two rice mutant shoots (*Oryza sativa ssp. Indica* cv. YueTai A (YTA) (Sterile Lines) (*O. sativa* YTA) and *Oryza sativa ssp. indica* cv. YueTai B (maintainer line) (*O. sativa* YTB)) were grown in the field for 3 months and harvested. Wild-type *O. sativa* L shoots, under three different light periods (all dark, 8 h light/16 h dark, 16 h light/8 h dark), were grown in a cultivation room at 25°C (night) and 30°C (day) for 2 weeks. The drought and control groups of *O. sativa* L were both germinated and grown in the cultivation room at 25°C (night) and 30°C (day) for 2 weeks. The seedlings grown without water were called the drought group, and the seedlings which were watered on time were called the control group. All plant materials were immediately frozen in liquid nitrogen after harvest and were then stored at -80°C.

### Preparation of hydrophilic magnetic sorbents

TiO_2_-coated magnetic hollow mesoporous silica spheres (MHMSS) were prepared according to a previously reported method with minor modification [33]. Briefly, CTAB (19.6 g) and Na_2_SiO_3_ · 9H_2_O (23.2 g) were dissolved in water (337 mL) to form a clear solution at 30°C. Then, ethyl acetate (35 mL) was quickly added, followed by vigorous stirring for 30 seconds. After standing at 30°C for 5 hours, the mixture was refluxed at 90°C for 48 hours. Finally, the mixture was filtered and washed several times with ethanol. The filtered HMSS was dried in a vacuum oven and then calcined at 550°C for 5 hours. Magnetic nanoparticles were introduced into the hollow core of HMSS through a vacuum impregnation of Fe(NO_3_)_3_. HMSS (2.4 g) was soaked in Fe(NO_3_)_3_ · 9 H_2_O aqueous solution (24 g/L, 200 mL). The suspension was heated in a microwave oven until boiling and then cooled in an ice water mixture, allowing the Fe^3+^ to enter the hollow core of the HMSS. The process was repeated several times until the water completely dried. Subsequently, the product was washed with 10 mL ethanol twice and dried again. The product was impregnated with 1 mL ethylene glycol up to incipient wetness. The impregnated sample was then subjected to heat treatment under nitrogen atmosphere at 450°C for 2 hours. Finally, TiO_2_ was loaded onto the obtained MHMSS through the liquid phase deposition method. MHMSS (2.0 g) was added into a solution (200 mL) containing 0.1 M (NH_4_)_2_TiF_6_ and 0.3 M H_3_BO_3_ in a PTFE container. After keeping under vacuum conditions for 1 h, the mixture was heated at 35°C for 12 h under continuous shaking. The resulting composite was washed with water thoroughly and dried at 60°C in a vacuum oven for 6 h. The resultant TiO_2_/MHMSS was obtained by heat treatment under nitrogen up to 300°C at the rate of 1 K/min and was then kept at 300°C for 2 h.

Nano-scale Fe_3_O_4_ was prepared through the solvothermal method according to a previously reported method [34]. FeCl_3_ · 6H_2_O (5.0 g) was dissolved in EG (100 mL) to form a clear solution. Then, NaAc (15.0 g) and ED (50 mL) were added to the solution. After vigorously stirring for 30 min, the homogeneous mixture was sealed in a Teflon-lined stainless-steel autoclave and was heated to 200°C for 8 hours. The product was magnetically collected and washed with water/ethanol several times and vacuum-dried at 60°C for 6 h.

### MSPE-ISD procedure for the determination of BRs in plant tissue

**Figure 8:**
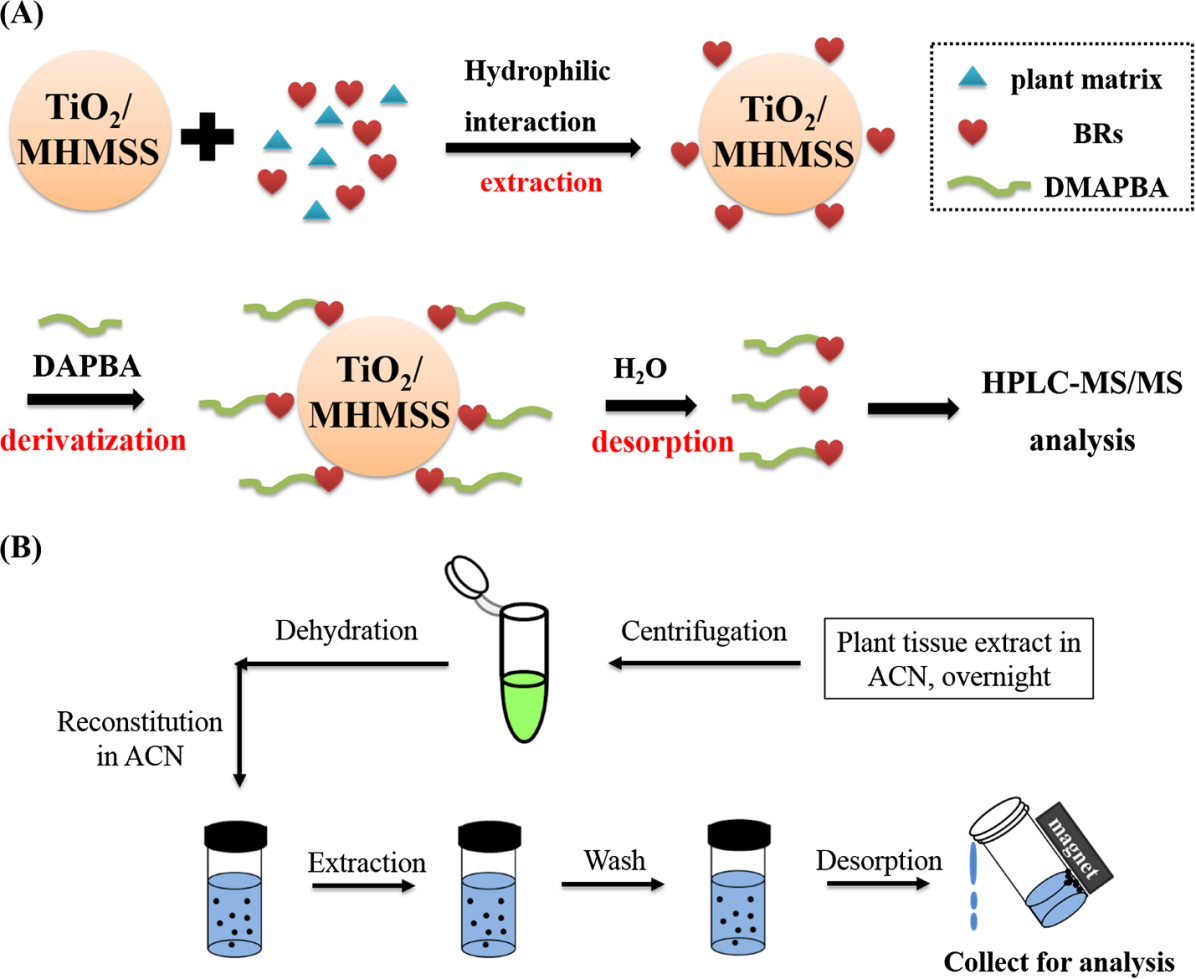
**Schematic illustration of the MSPE-ISD method (A) and sample pretreatment strategy (B) for BRs in plant tissues.**

The schematic illustrations of MSPE-ISD (A) and the sample pretreatment strategy (B) are depicted in Figure 8. Plant tissue (100 mg fresh weight (FW)) was smashed with a mortar and pestle in liquid nitrogen. The powdered sample was extracted at –20°C overnight with acetonitrile (1 mL) containing [^2^H_3_]BL and [^2^H_3_]CS (0.4 ng each) as IS for quantification. After centrifugation at 3,500 g for 10 min, the supernatant was collected in a 1.5-mL vial followed by evaporation to dryness under a mild nitrogen gas stream. The residue was re-dissolved with acetonitrile (1 mL) for the following MSPE-ISD process.

TiO_2_/MHMSS (50 mg) was added to a 15-mL glass vial and activated with acetonitrile before use. Subsequently, the aforementioned plant extract (1 mL) was added into the vial and vortexed vigorously for 30 seconds to form a homogenous dispersive solution. The supernatant was separated and discarded by applying a magnet. Acetonitrile (1 mL) was added to wash the residual matrix interferences on the surface with 30 seconds of vortexing and was then disposed of. The washing process was repeated twice. Subsequently, DMAPBA-acetonitrile solution (500 μg/mL, 1 mL) was added to the vial for ISD by vortexing for 30 seconds. Finally, water (0.5 mL) was added to the mixture solution to elute BR derivatives from the sorbents by 30 seconds of vortexing. The desorption solution was magnetically separated and evaporated to dryness under a mild nitrogen gas flow at 35°C. The residue was dissolved in acetonitrile/H_2_O (50 μL, 1/1 v/v), and then 20 μL was used for the analysis by UPLC-ESI-MS/MS.

### UPLC-ESI-MS/MS analysis

The mass spectrometry analysis was performed on a UPLC-ESI (+)-MS/MS system consisting of a Shimadzu LC-30AD HPLC system (Tokyo, Japan) with two 30AD pumps, an SIL-30AC auto sampler, a CTO-30A thermostat column compartment, a DGU-20A_5R_ degasser, and a Shimadzu MS-8040 mass spectrometer (Tokyo, Japan) with an electrospray ionization source (Turbo Ionspray). The separation of BRs was achieved on a Shim-pack ODS column (75 × 2.0 mm id, 1.6 μm, Shimadzu, Tokyo, Japan). The column oven temperature was set at 40°C. Mobile phases A and B were 0.1% formic acid in water and acetonitrile, respectively. An isocratic elution of 85% B at 0.2 mL/min for 7 minutes was employed. The injection volume was 20 μL.

All BRs were quantified by multiple reaction monitoring (MRM) in the positive mode. The optimal ESI source conditions were as follows: DL temperature 250°C, heat block temperature 400°C, nebulizing gas 3 L/min and drying gas 15 L/min. The MRM mass spectrometric parameters are summarized in Table 7. Data were acquired by Labsolutions software (version 5.53 sp2, Shimadzu, Tokyo, Japan).

**Table 7:** **Optimized MRM parameters of seven BR derivatives by UPLC-ESI-MS/MS**

Analyte	Quantification					Confirmation				
	Q1 (m/z)	Q3 (m/z)	Q1 pre bias/V	CE	Q3 pre bias/V	Q1(m/z)	Q3(m/z)	Q1 pre bias/V	CE	Q3 pre bias/V
28-norBL	596.4	190.1	-30	-44	-20	596.4	246.1	-30	-34	-17
BL	610.4	190.2	-32	-41	-13	610.4	122.1	-32	-40	-24
28-homoBL	624.4	190.2	-32	-41	-20	624.4	418.0	-32	-39	-23
28-norCS	580.4	190.1	-30	-49	-21	580.4	562.4	-30	-30	-28
CS	594.4	190.1	-30	-44	-20	594.4	576.4	-30	-32	-22
^2^H_3_BL	613.4	190.4	-34	-51	-13	613.4	345.4	-34	-38	-16
^2^H_3_CS	597.4	194.4	-32	-46	-13	597.4	579.6	-32	-32	-22
